# Isoporous Membranes from Novel Polystyrene-*b*-poly(4-vinylpyridine)-*b*-poly(solketal methacrylate) (PS-*b*-P4VP-*b*-PSMA) Triblock Terpolymers and Their Post-Modification

**DOI:** 10.3390/polym12010041

**Published:** 2019-12-26

**Authors:** Sarah Saleem, Sofia Rangou, Clarissa Abetz, Volkan Filiz, Volker Abetz

**Affiliations:** 1 Helmholtz-Zentrum Geesthacht, Institute of Polymer Research, Max-Planck-Str.1, 21502 Geesthacht, Germany; sarah.saleem@hzg.de (S.S.); sofia.rangou@hzg.de (S.R.); clarissa.abetz@hzg.de (C.A.); volkan.filiz@hzg.de (V.F.); 2 Institute of Physical Chemistry, Universität Hamburg, Martin-Luther-King-Platz 6, 20146 Hamburg, Germany

**Keywords:** triblock terpolymer, self-assembly, isoporous membrane, non-solvent-induced phase separation (NIPS), ultrafiltration, acidic hydrolysis

## Abstract

In this paper, the formation of nanostructured triblock terpolymer polystyrene-*b*-poly(4-vinylpyridine)-*b*-poly(solketal methacrylate) (PS-*b*-P4VP-*b*-PSMA), polystyrene-*b*-poly(4-vinylpyridine)-*b*-poly(glyceryl methacrylate) (PS-*b*-P4VP-*b*-PGMA) membranes via block copolymer self-assembly followed by non-solvent-induced phase separation (SNIPS) is demonstrated. An increase in the hydrophilicity was observed after treatment of non-charged isoporous membranes from PS-*b*-P4VP-*b*-PSMA, through acidic hydrolysis of the hydrophobic poly(solketal methacrylate) PSMA block into a hydrophilic poly(glyceryl methacrylate) PGMA block, which contains two neighbored hydroxyl (–OH) groups per repeating unit. For the first time, PS-*b*-P4VP-*b*-PSMA triblock terpolymers with varying compositions were successfully synthesized by sequential living anionic polymerization. Composite membranes of PS-*b*-P4VP-*b*-PSMA and PS-*b*-P4VP-*b*-PGMA triblock terpolymers with ordered hexagonally packed cylindrical pores were developed. The morphology of the membranes was studied with scanning electron microscopy (SEM) and atomic force microscopy (AFM). PS-*b*-P4VP-*b*-PSMA triblock terpolymer membranes were further treated with acid (1 M HCl) to get polystyrene-*b*-poly(4-vinylpyridine)-*b*-poly(glyceryl methacrylate) (PS-*b*-P4VP-*b*-PGMA). Notably, the pristine porous membrane structure could be maintained even after acidic hydrolysis. It was found that membranes containing hydroxyl groups (PS-*b*-P4VP-*b*-PGMA) show a stable and higher water permeance than membranes without hydroxyl groups (PS-*b*-P4VP-*b*-PSMA), what is due to the increase in hydrophilicity. The membrane properties were analyzed further by contact angle, protein retention, and adsorption measurements.

## 1. Introduction 

Block copolymers have been demonstrated as promising precursors for the fabrication of highly ordered nanoporous structures. One of the ubiquitous features of block copolymers is their ability to form a plethora of nanoscale ordered structures. Various techniques to control synthesis, along with theoretical models, make it possible to define precisely the morphology and size of the microdomains [1,2,3,4,5,6,7,8]. ABC triblock terpolymers (triblock terpolymers) have received much attention due to a broadly enhanced spectrum of applications [9,10,11,12,13,14]. Self-assembly of block copolymers in solution is influenced by the interaction of the blocks among themselves, as well as the interactions of the blocks with the solvent or solvent mixture, which is more complex in case of triblock terpolymers compared to diblock copolymers. Triblock terpolymers are exciting materials to explore for membrane applications because of their extended possibilities to tune their properties compared to diblock copolymers. Properties of block copolymers for specific applications can be tailored by the introduction of functional components and subsequent chemical modification [15,16,17,18]. 

In the field of water purification and protein separation, block copolymer filtration membranes attracted some attention [19,20,21,22]. Foulants like proteins, emulsified oils, microorganisms, and a fraction of natural organic matters can be separated by polymeric membranes, as a higher affinity for adhesion is observed for hydrophobic than hydrophilic membranes. Poor antifouling properties are mainly caused by the hydrophobic behavior of membranes surfaces. Therefore, many surface modifications focus on hydrophilizing a hydrophobic membrane surface as it is hypothesized that hydrophilic surfaces are prone to tightly bind a layer of water, which frustrates the deposition of foulants from aqueous media [23,24,25]. Sharp molecular weight cut-offs (MWCO) in combination with high flux on a large scale by using simple techniques is the goal of membrane technology to make membrane-based separations efficient [26,27,28].

A combination of self-assembly and non-solvent-induced phase separation (SNIPS) leads to both effective and scalable processes to produce integral asymmetric membranes [29,30,31]. The approach of SNIPS for isoporous membrane formation was applied for the first time to the amphiphilic diblock copolymer polystyrene-*block*-poly(4-vinyl pyridine) (PS-*b*-P4VP) [32]. It is the most widely studied diblock copolymer to date for the fabrication of integral asymmetric membranes. However, the phase inversion technique was successfully applied also to other diblock copolymer systems polystyrene-*b*-poly(ethylene oxide) (PS-*b*-PEO) [33,34], polystyrene-*b*-poly(2-vinylpyridine) (PS-*b*-P2VP) [35], polystyrene-*b*-poly(2-hydroxyethyl methacrylate) (PS-*b*-PHEMA) [36,37], polystyrene-*b*-poly(methyl methacrylate) (PS-*b*-PMMA) [38], poly(α-methylstyrene)-*b*-poly(4-vinylpyridine) (PαMS-*b*-P4VP) [39], and also triblock terpolymer systems such as polyisoprene-*b*-polystyrene-*b*-poly(4-vinylpyridine) (PI-*b*-PS-*b*-P4VP) [15], polyisoprene-*b*-polystyrene-*b*-poly(N,N-dimethylacrylamide) (PI-*b*-PS-*b*-PDMA) [40], and polystyrene-*b*-poly(2-vinylpyridine)-*b*-poly(ethylene oxide) (PS-*b*-P2VP-*b*-PEO) [41]. Although many different block copolymers were used for the SNIPS process to generate isoporous membranes featuring comparably polar surfaces, no approaches for the preparation of such membranes were reported using two neighbored hydroxyl groups (–OH) containing block copolymers. In our group, hydroxyl moieties were introduced into the isoporous standing cylinders after post-modification of PS-*b*-P4VP based membranes by applying atom transfer radical polymerization protocols after polydopamine coating [42]. As a result, the authors found increased hydrophilicity and enhanced heat resistance of the obtained membranes.

In this study, the hydrophobic PSMA block is introduced as a third block to the PS-*b*-P4VP system with the aim to broaden the window of possible post-modification and at the same time to improve the antifouling behavior of the membrane. Acidic hydrolysis of the ketal group of hydrophobic PSMA results in hydrophilic PGMA, containing two hydroxyl groups per repeating unit of the polymer. Because of its larger hydrophilicity, PGMA has the potential to replace the less hydrophilic 2-hydroxyethyl methacrylate (HEMA) in many fields. It has also been reported as a material for ultrafiltration barriers mimicking the natural membranes in kidneys [43]. Poly(isopropylidene glycerol methacrylate), commonly known as poly(solketal methacrylate), is one of those polymers not yet studied widely in the context of block copolymer membranes produced via the SNIPS process [44,45,46,47]. Recently, we reported a detailed comparison of double hydrophobic PS-*b*-PSMA and amphiphilic PS-*b*-PGMA diblock copolymer membranes, where the influence of the hydrophobic or hydrophilic nature of the second block was analyzed by water flux and contact angle measurements. In contrary to the present manuscript, the post-modification of PSMA was performed in the polymer solution [48]. Here we report for the first time the synthesis of a series of amphiphilic triblock terpolymers PS-*b*-P4VP-*b*-PSMA by living anionic polymerization, followed by the exploration of triblock terpolymer membrane formation in varying polymer concentrations, solvent compositions, and evaporation times. This is followed by a comparative characterization of both (PS-*b*-P4VP-*b*-PSMA) and (PS-*b*-P4VP-*b*-PGMA) triblock terpolymer membranes.

## 2. Materials and Methods 

### 2.1. Size Exclusion Chromatography (SEC)

The molecular weights of the polystyrene precursor and molecular weight distribution of the block copolymer were determined by gel permeation chromatography calibrated with PS standards. The measurements were performed at 50 °C in *N*,*N*-dimethylacetamide (Sigma-Aldrich, Schnelldorf, Germany) with addition of lithium (Sigma-Aldrich, Schnelldorf, Germany)chloride using PSS GRAM columns (GRAM precolumn (dimensions, 8.50 mm), GRAM column (porosity, 3000 A; dimensions; 8.30 mm; particle size 10 μm), and GRAM column (porosity, 1000 Å; dimension, 8.30 mm; particle size, 10 μm)) (Polymer Standards Service, Mainz, Germany) at a flow rate of 1.0 mL min^−1^ (VWR-Hitachi 2130 pump (Hitachi, Tokyo, Japan)). A Shodex RI-101 refractive index detector (Shodex, Kanagawa, Japan) with a polystyrene calibration was used.

### 2.2. Nuclear Magnetic Resonance Spectroscopy (NMR)

The triblock terpolymers were analyzed by proton nuclear magnetic resonance spectroscopy (^1^H-NMR). ^1^H-NMR measurements were performed on a Bruker Ascend 500 NMR spectrometer (500 MHz) (Bruker BioSpin GmbH, Rheinstetten, Germany) using CDCl_3_ (Sigma-Aldrich, Schnelldorf, Germany) and DMF-*d*7 (Sigma-Aldrich, Schnelldorf, Germany) as solvent at room temperature.

### 2.3. Scanning Electron Microscopy (SEM)

Scanning electron microscopy (SEM) of the membranes was carried out on a LEO Gemini 1550 VP (Zeiss, Oberkochen, Germany) at a voltage of 3 or 5 kV. The samples were coated with 2.0 nm platinum using a coating device Bal-tec MED 020 (Bal-tec/Leica Microsystems GmbH, Wetzlar, Germany). Cross-sections of the membranes were prepared while dipping the membranes in isopropanol, freezing in liquid nitrogen, and breaking. Average pore size values were determined using the software Analysis (Olympus Soft Imaging Solutions GmbH, Münster, Germany) based on the SEM results. 

### 2.4. Transmission Electron Microscopy (TEM)

Transmission electron microscopy (TEM) was carried out with a Tecnai G2 F20 (Thermo Fisher Scientific (formerly FEI), Eindhoven, The Netherlands) operated at 120 kV in bright-field mode. Thin sections (thin section thickness: 50 nm) were cut using a Leica Ultramicrotome EM UCT (Leica Microsystems, Wetzlar, Germany) equipped with a diamond knife (Diatome AG, Biel, Switzerland). 

### 2.5. Atomic Force Microscopy (AFM)

The surfaces of the membranes were imaged with a Bruker MultiMode 8 (Bruker Nanosurfaces, Karlsruhe, Germany) in Peak Force QNM (Quantitative Nanomechanical Mapping) mode at room temperature. For measurements in the dry state, ScanAsyst-Air probes and, for measurements in liquid, ScanAsyst-Fluid+ probes in a liquid cell were used. For the analysis, the software NanoScope Analysis 1.5 (Bruker Nanosurfaces, Karlsruhe, Germany) was used.

### 2.6. Contact Angle Measurements

A Drop Shape Analyzer DSA 100 (KRÜSS GmbH, Hamburg, Germany) was used for the measurement of the dynamic contact angle. The measurements were carried out with a sessile droplet of 2 μL ultrapure water at room temperature.

### 2.7. Water Flux and Retention Measurements

Water flux and retention measurements were performed using a stirred test cell (EMD Millipore^TM^ XFUF04701) (Merck Millipore, Darmstadt, Germany) in dead-end mode at a trans-membrane pressure (TMP) of 2 bar at room temperature. The membrane area was 1.8 cm^2^. These studies were conducted employing demineralized water with an electrical conductivity of ≈0.055 µScm^−1^. BSA (bovine serum albumin) (Sigma-Aldrich, Schnelldorf, Germany) dissolved in PBS buffer solution at a concentration of 1 g/L was employed for the retention measurements. The concentrations of BSA solutions were measured at the wavelength of 280 nm with a UV–Vis spectrophotometer (GENESYS 10S, Thermo Fisher Scientific, Bremen, Germany). The BSA retention was calculated by the following equation:

R = (1 − c(p)/c(f)) × 100

where c(p) and c(f) represent the BSA concentrations (g/L) in the permeation and feed.

### 2.8. Static Protein Adsorption

The protein adsorption capacity of the membranes was evaluated through static protein adsorption experiments using hemoglobin solutions with a concentration of 1.0 g/L in a PBS buffer solution (10 mM PBS, 0.9 wt. % NaCl) (Sigma-Aldrich, Schnelldorf, Germany). To ensure complete wetting of the membrane structure with the protein solution, all membrane samples were immersed in PBS buffer and washed twice. Subsequently, 2 mL of the protein solution was placed on each sample in a closed vial. To reach equilibrium, the samples were shaken for 24 h at 90 rpm and at 25 °C. After that, each membrane sample was rinsed two times with 2 mL PBS buffer for 10 min. The protein adsorption values were calculated as follows:

Protein adsorption = m_0_ − (m_1_ + m_w1_ + m_w2_)/A_membrane_

where m_0_ is mass of the protein before the adsorption experiment and m_1_ is the protein mass after the adsorption experiment, while m_w1_ and m_w2_ are the protein masses in the washing solutions. The adsorption value is related to the membrane surface area (A_membrane_). The concentrations of the protein were determined by UV–VIS spectroscopy (GENESYS 10S, Thermo Fisher Scientific) at a wavelength of λ = 280 nm.

### 2.9. Synthesis of Polystyrene-block-poly(4-vinylpyridine)-block-poly(solketal methacrylate) Triblock Terpolymers

The linear PS-*b*-P4VP-*b*-PSMA triblock terpolymers were synthesized by sequential living anionic polymerization in a Schlenk line apparatus using high vacuum (10^−7^–10^−8^ mbar) and Argon supply (Argon 7.0, Linde AG, Pullach, Germany). Styrene (Sigma-Aldrich, Schnelldorf, Germany, 99%) was distilled from di-*n*-butylmagnesium (Sigma-Aldrich, Schnelldorf, Germany, 1.0 M solution in heptane) under high vacuum. 4-Vinylpyridine (4VP) (Sigma-Aldrich, Munich, Germany) was distilled once from calcium hydride and twice from ethyl aluminum dichloride (Sigma-Aldrich, Schnelldorf, Germany). Solketal methacrylate (BASF SE Corporation, Ludwigshafen, Germany) was distilled twice from calcium hydride (Sigma-Aldrich, Munich, Germany). The reaction solvent was THF, distilled and titrated with sec-butyl lithium (*sec*-BuLi) (Sigma-Aldrich, Schnelldorf, Germany, 1.4 M solution in cyclohexane). The synthetic procedure involves first the anionic polymerization of styrene in THF with *sec*-BuLi at −78 °C. After two hours an aliquot was taken for SEC analysis, followed by the addition of 4-vinylpyridine and the solution was stirred overnight. Another aliquot was taken from the polymerization reactor and was terminated with degassed methanol for molecular characterization. PS-*b*-P4VP macroinitiator was end-capped with 1,1-diphenylethylene (Sigma-Aldrich, Schnelldorf, Germany) by maintaining the temperature at −30 °C for half an hour. Afterward, purified solketal methacrylate was added to the mixture and the temperature was decreased again to −78 °C. After two hours, the polymerization was quenched with degassed methanol/acetic acid (9:1). THF was removed under reduced pressure and the polymer was precipitated in a water/methanol mixture (80/20 *v*/*v*). The final product was dried in a vacuum oven for 48 h at 50 °C to give a colorless powder.

The number average molecular weight (*M*_n_) and dispersity index (Ð) of the intermediate products and the final triblock terpolymers were determined by SEC. The chemical composition of the triblock terpolymers was determined using ^1^H-NMR. 

Molecular weight of the triblock terpolymers was determined by combining the (*M*_n_) value for the PS precursor blocks (SEC) with the blocks weight percentages calculated by ^1^H-NMR (Table 1). 

### 2.10. Membrane Fabrication

Membranes were prepared by casting concentrated terpolymer solutions (21–24 wt. %) with a doctor blade having a gap of 200 µm on polyester non-woven followed by a specific evaporation step (time) and finally immersion of the film into the precipitation bath. All solvents used for block copolymer solution formation are miscible with water and chosen based on solubility parameters.

### 2.11. Post-Modification of PS-b-P4VP-b-PSMA Triblock Terpolymer and Membranes 

Isoporous membranes obtained from PS_71_-*b*-P4VP_26_-*b*-PSMA_3_^145^ and PS_70_-*b*-P4VP_25_-*b*-PSMA_5_^143^, as well as the powder of PS_71_-*b*-P4VP_17_-*b*-PSMA_12_^91^ were treated with 1 M HCl (Thermo Fisher Scientific, Karlsruhe, Germany) solution at 50 °C for 3 days to remove the acetonide moiety of PSMA blocks. The acid-treated membranes were further dipped in 0.1 M NaOH (Thermo Fisher Scientific, Kandel, Germany) solution for 45 min to completely deprotonate the quaternized P4VP blocks. Similarly, the triblock terpolymer was stirred in 0.1 M NaOH solution for 45 min. Finally, the two membranes and the other triblock terpolymer were treated with deionized water and dried in the vacuum oven at 50 °C. The procedure was monitored by ^1^H-NMR to ensure the removal of the isopropylidene acetal group. After each step, the membrane selective layer, or the polymer in the latter case, was re-dissolved in deuterated solvents and spectra were recorded. Quaternization of the P4VP was monitored by the solubility of the polymer in CDCl_3_ (quaternized polymers were not dissolved in CDCl_3_).

## 3. Results

**Figure 1:**
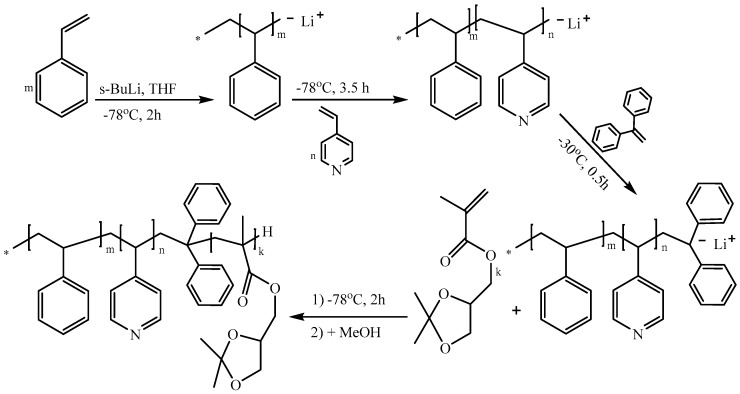
Synthetic route leading to PS-*b*-P4VP-*b*-PSMA by sequential anionic polymerization of styrene, 4-vinyl pyridine, and solketal methacrylate.

A three-step sequential living anionic copolymerization procedure was employed for the synthesis of PS-*b*-P4VP-*b*-PSMA triblock terpolymers. The polymerization route is depicted in Figure 1. 

After hydrolysis of the PSMA block into a PGMA block, two hydroxyl groups will be available for post-modification. Therefore, compared to PS-*b*-P4VP membranes, PS-*b*-P4VP-*b*-PGMA membranes could allow various possibilities of post-modification. Additionally, when compared to PS-*b*-P4VP membranes, PS-*b*-P4VP-*b*-PGMA membranes would potentially increase the resistance toward fouling.

### 3.1. Bulk Morphology of the Triblock Terpolymers 

**Figure 2:**
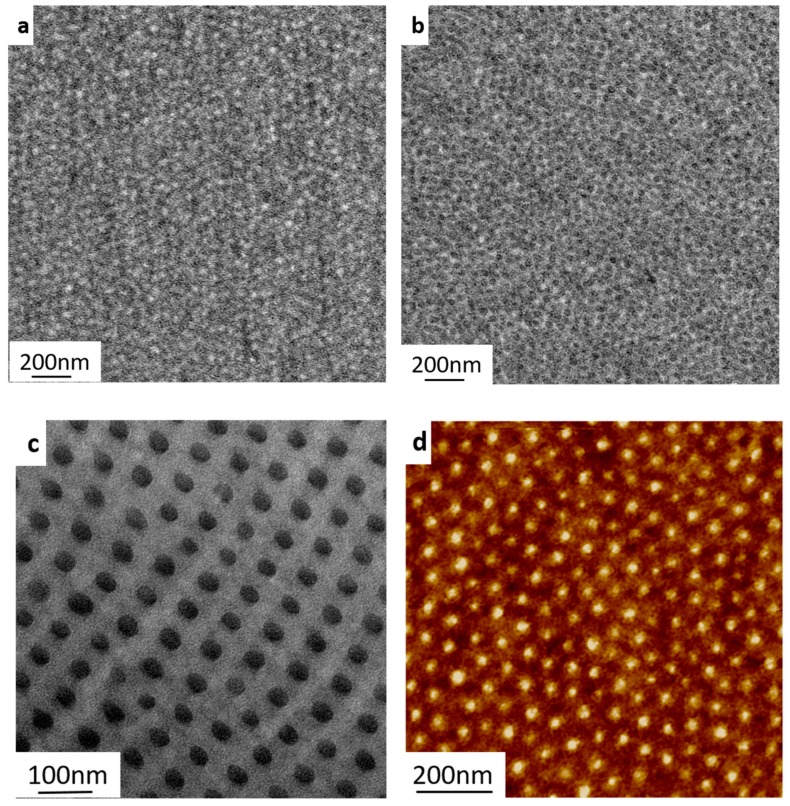
TEM micrographs of PS-*b*-P4VP-*b*-PSMA of different molecular weights cast from chloroform. (**a**) PS_71_-*b*-P4VP_17_-*b*-PSMA_12_^91^ stained with RuO_4_. (**b**) PS_71_-*b*-P4VP_17_-*b*-PSMA_12_^91^ stained with RuO_4_ and I_2_ (**c**). PS_71_-*b*-P4VP_26_-*b*-PSMA_3_^145^ stained with I_2_ (**d**). AFM height image (in tapping mode) of the film surface of the asymmetric PS_71_-*b*-P4VP_26_-*b*-PSMA_3_^145^ triblock terpolymer.

Annealed films obtained from solution casting were used to study the bulk morphology of the triblock terpolymers. TEM analysis of two PS-*b*-P4VP-*b*-PSMA triblock terpolymers (PS_71_-*b*-P4VP_26_-*b*-PSMA_3_^145^, PS_71_-*b*-P4VP_17_-*b*-PSMA_12_^91^) was performed by preparing ultrathin sections of the respective triblock terpolymer film. For this purpose, a 7.5 wt. % solution of polymer was prepared in CHCl_3,_ which is a common rather non-selective solvent for all the three blocks. The solution dried for a week in a porcelain crucible under a constant vapor atmosphere in a desiccator. The films were further annealed slowly from room temperature to 150 °C. Figure 2a shows the TEM micrograph of a PS_71_-*b*-P4VP_17_-*b*-PSMA_12_^91^ triblock terpolymer film in which only the PS block was stained with RuO_4_. In this case, no typical or well-defined morphology could be evidenced, however, some bright domains of PSMA could be observed in the darker PS matrix. Later, the P4VP blocks of the same ultrathin section were stained with iodine vapor. The result is depicted in Figure 2b. In the presence of iodine staining, the PSMA block remains unstained. Gray features demonstrate the PS block, which are distributed in the darker phase constituted by P4VP. Regular hexagonally packed cylindrical structures predominate in PS_71_-*b*-P4VP_26_-*b*-PSMA_3_^145^ with no clear differentiation of the phase composition as shown in Figure 2c.

Figure 2d shows an AFM image of a thin film of PS_71_-*b*-P4VP_26_-*b*-PSMA_3_^145^ prepared by spin coating on a silicon wafer from a solution in chloroform and the long-range ordered morphology was confirmed.

### 3.2. Preparation of the Membranes by SNIPS 

Among the parameters influencing the nanostructure of a membrane prepared by SNIPS, the (mixed) solvent interactions to different blocks and the concentration of the polymer are the most important ones [49,50]. 

A rational design of the selective solvent mixture plays a decisive role in the SNIPS process for the desired morphology of block copolymer membranes. Solutions of block copolymer with binary or ternary mixtures of solvents varying from volatile to non-volatile were prepared. The volatile solvents THF and acetone are suitable for both PS and PSMA blocks, whereas DMF is the preferred solvent for P4VP blocks according to the solubility parameters of the solvents and blocks of the block copolymers. In this study, the Hoy method [51] was used to calculate solubility parameters of PSMA and PGMA homopolymers, as displayed in Table 2. The parameters to obtain the desired integral asymmetric structure with cylindrical pores oriented perpendicular to the membrane surface were optimized by trial and error.

**Figure 3:**
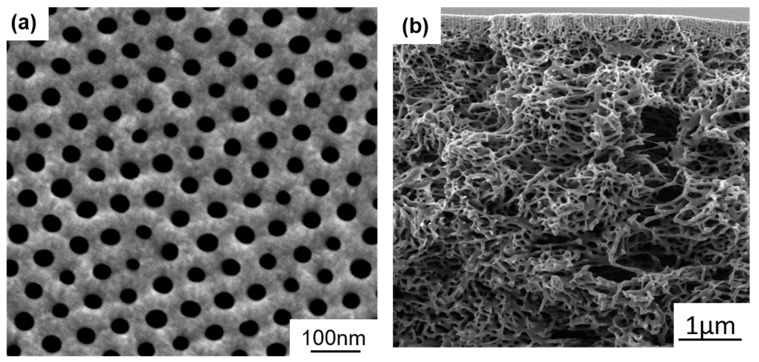
SEM images of (**a**) top view and (**b**) cross-section of PS_71_-*b*-P4VP_26_-*b*-PSMA_3_^145^ membranes cast from 23 wt. % solution in a THF/DMF/acetone: 50/30/20 wt. %. Evaporation time was 10 s before immersion in water.

Initially, we prepared membranes using different concentrations of binary solvent mixtures THF/DMF: 50/50, 60/40, 70/30 (*w*/*w*), however, the morphologies of the PS_71_-*b*-P4VP_26_-*b*-PSMA_3_^145^ membranes did not show ordered pore structures, but rather dense regions with random macropores were obtained (Supporting Information Figure S1). Therefore, two types of ternary solvent mixtures THF/DMF/DOX and THF/DMF/acetone were used for membrane casting. Self-assembly of the triblock terpolymer was not successful in different compositions of THF/DMF/DOX (Supporting Information Figure S2). However, the addition of acetone to the THF/DMF mixture directs the self-assembly of the triblock terpolymer into highly ordered hexagonally packed cylinders with perpendicular orientation. Different combinations of THF/DMF/acetone were examined, but only the mixture of THF/DMF/acetone (50/30/20 wt. %) leads to the desired nanostructure containing narrow pore size distribution with 10 s evaporation time, as shown in Figure 3a. THF and acetone are selective volatile solvents for PS and PSMA while DMF is a more selective and much less volatile solvent for P4VP. The abrupt change in the polymer solution concentration due to the fast evaporation of acetone and THF favors the perpendicular ordering of the microdomains, and this differs from the solvent mixture containing less volatile DOX instead of acetone. Phillip et al. [30] reported that fast evaporation conditions on the top layer can form perpendicular cylinders templated by the copolymer self-assembly, while the underlying porous structure is controlled by the polymer precipitation. The cross-section of the triblock terpolymer membrane was imaged by SEM in Figure 3b. The mean pore diameter of the integral asymmetric membrane is 26 ± 3 nm. It is advantageous to have a volatile co-solvent to study the effect of evaporation time for PS_71_-*b*-P4VP_26_-*b*-PSMA_3_^145^ membranes. By increasing the evaporation time to 20 s, membrane regularity was partially destroyed and a dense structure was formed predominately with only few open pores. Due to the longer evaporation time, the very well-ordered hexagonal cylindrical structure collapses. The lying cylindrical structure is much less porous and thus retards the solvent/non-solvent exchange. (Supporting Information Figure S3). It is observed that in-diffusion of non-solvent and out-diffusion of co-solvent becomes more difficult by increasing the polymer concentration of the casting solution from 21–24 wt. %. The concentration of PS_71_-*b*-P4VP_26_-*b*-PSMA_3_^145^ was varied by keeping all the other parameters constant. Membranes with a top isoporous layer (with cylindrical channels of approximately 220 nm length), followed by the spongy structure underneath, were obtained. High viscosity of the more concentrated casting solutions results from entanglements of the polymer chains, and perhaps influences the final membrane morphology (Supporting information Figure S4).

### 3.3. Post-Modification of the Membranes

**Figure 4:**
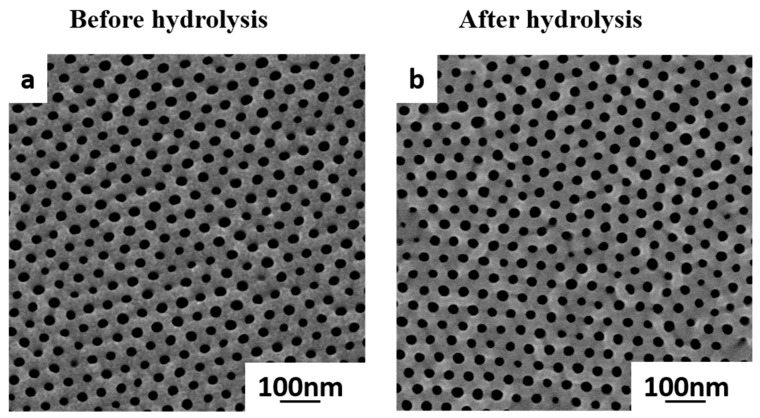
(**a**) SEM images of pristine PS_71_-*b*-P4VP_26_-*b*-PSMA_3_^145^ membrane before acidic hydrolysis and (**b**) PS_71_-*b*-P4VP_26_-*b*-PGMA_3_^145^ membrane.

Our next aim was the conversion of the hydrophobic (PSMA) block of the pristine (PS-*b*-P4VP-*b*-PSMA) membranes into highly hydrophilic (PGMA) block via acidic hydrolysis. This was achieved in solid state (membrane) by using hydrochloric acid (1 N) while the original integral asymmetric isoporous structure of the membrane is preserved (Figure 4). 

**Figure 5:**
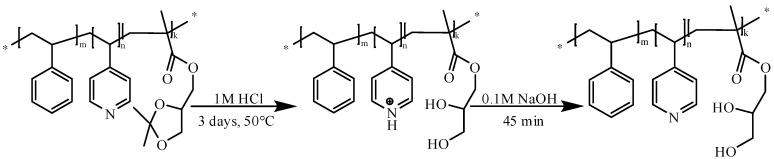
The deprotection reaction of the ketal-PSMA moiety.

The complete acidic hydrolysis of the ketal moiety of the PSMA block shown in the following Figure 5. 

**Figure 6:**
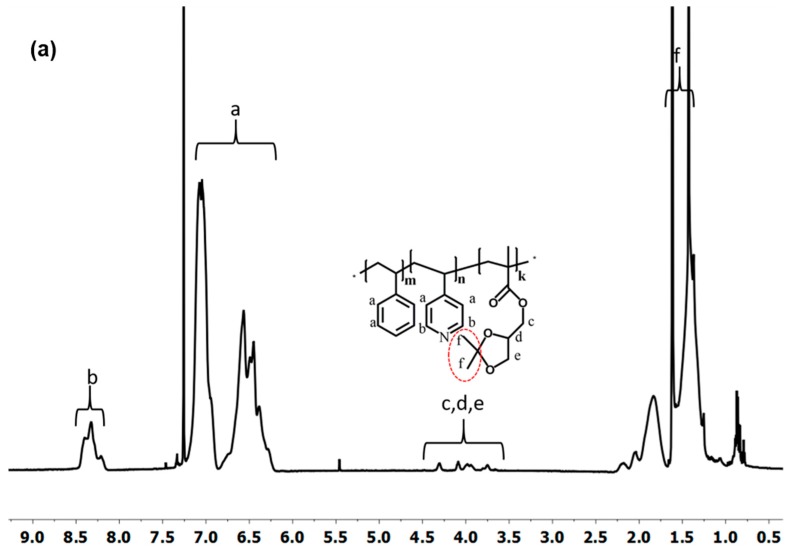

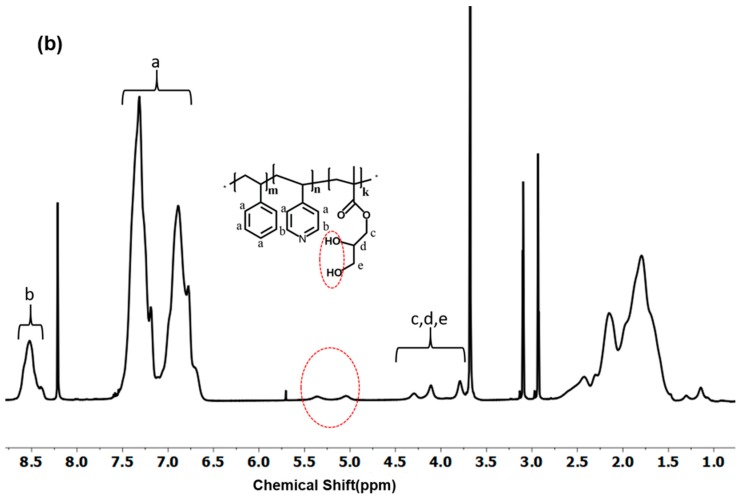
^1^H-NMR spectrum (in CDCl_3_) of the linear triblock terpolymer re-dissolved membranes (**a**) PS_71_-*b*-P4VP_26_-*b*-PSMA_3_^145^ before and (**b**) after hydrolysis in DMF-*d*7.

A comparison of ^1^H-NMR spectra of PS_71_-*b*-P4VP_26_-*b*-PSMA_3_^145^ polystyrene-*b*-poly(4-vinylpyridine)-*b*-poly(solketal methacrylate) and polystyrene-*b*-poly(4-vinylpyridine)-*b*-poly(glyceryl methacrylate) PS_71_-*b*-P4VP_26_-*b*-PGMA_3_^145^ is shown in Figure 6a,b. The appearance of new signals at 5.3 and 5.5 ppm corresponds to two hydroxyl groups (–OH) of GMA units, whereas the signals at 3.7–4.2 ppm represent five protons of the solketal moiety, which are shifted up field due to the replacement of the carbon atom with a hydrogen atom. A PS-*b*-P4VP-*b*-PSMA membrane was also protonated by HCl during the acidic hydrolysis of the ketal moieties of the PSMA block, resulting in temporarily charged PS-*b*-PQ4VP-*b*-PGMA (PQ4VP stands for quarternized P4VP) membranes because of the 4-vinyl pyridine moiety quaternization. However, the temporary quarternized P4VP block was deprotonated successfully by dipping the membrane in 0.1 M sodium hydroxide solution. 

The highly isoporous structure of the prepared membranes was intact even after all the modifications. SEM images of the membranes from the PS_70_-*b*-P4VP_25_-*b*-PSMA_5_^143^ triblock terpolymer are provided in the Supporting Information (Figure S5). The membranes cast by SNIPS from the solution of the PS_71_-*b*-P4VP_17_-*b*-PSMA_12_^91^ (larger PSMA block) exhibited a dense structure even if the same composition of solvents was used (Supporting Information Figure S6a). Based on previous findings [41,48], this is probably due to the shorter hydrophilic P4VP block, which cannot compensate for the hydrophobic nature of the PSMA block. Apparently, the amphiphilic behavior plays a crucial role in the self-assembly and pore formation during the phase inversion process. After the acidic hydrolysis of PS_71_-*b*-P4VP_17_-*b*-PSMA_12_^91^ triblock terpolymer, the amphiphilicity of the whole polymer increased due to the appearance of two hydroxyl groups in PS_71_-*b*-P4VP_17_-*b*-PGMA_12_^91^, resulting into a hexagonally oriented cylindrical porous membrane (Supporting information Figure S6b). It is assumed that the hydrophilic short block at the end of the pore-forming block (P4VP) enhances the formation of a good membrane structure, while the addition of a hydrophobic block tends to suppress the formation of a porous membrane structure. Only if the P4VP block is large enough can it overcome the influence of the hydrophobic PSMA block and a membrane with highly oriented pores can be obtained. This is in agreement with our previous results for a PS-*b*-PSMA and the corresponding hydrolyzed PS-*b*-PGMA diblock copolymer, where only the latter one yielded isoporous membranes by applying SNIPS [48].

### 3.4. Contact Angle Measurements

**Figure 7:**
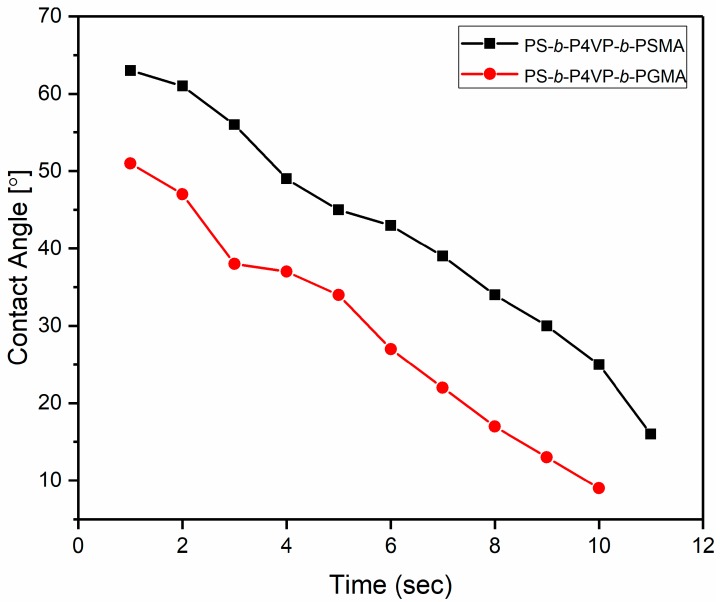
Dynamic contact angle measurements of water droplets (2 µL each) onto the PS_71_-*b*-P4VP_26_-*b*-PSMA_3_^145^ (**black squares**) and PS_71_-*b*-P4VP_26_-*b*-PGMA_3_^145^ (**red circles**) membranes.

The hydrophilicity of the membrane is characterized by water contact angle. The sessile drop method was used to investigate the dynamic contact angle of PS_71_-*b*-P4VP_26_-b-PSMA_3_^145^ and PS_71_-*b*-P4VP_26_-*b*-PGMA_3_^145^ membranes. Figure 7 shows the contact angles or sinking of a water droplet with time on/into the membrane surfaces before and after hydrolysis of PSMA blocks. The detailed values of contact angle measurements are given in Table S1 (Supporting Information). It can be seen that the water contact angle of PS_71_-*b*-P4VP_26_-*b*-PSMA_3_^145^ membrane surface is definitely higher as compared to the PS_71_-*b*-P4VP_26_-*b*-PGMA_3_^145^ membranes. The lower contact angle value for PS_71_-*b*-P4VP_26_-*b*-PGMA_3_^145^ membranes surfaces indicates the presence of hydroxyl (–OH) groups on the surface or PGMA block covers the inner surface of the pores. In general, contact angle measurements are influenced by the chemical composition of the surface of the membrane and membrane porosity. The higher water contact angle and a lower rate of sinking of a water droplet on the pristine (PS-*b*-P4VP-*b*-PSMA) membrane surface can be explained by the hydrophobic nature of the PSMA block. In the case of the PS-*b*-P4VP-*b*-PGMA membrane, the presence of (–OH) groups in PGMA end blocks effectively compete with water by hydrogen bonding and van der Waals interactions lead to lower contact angle [53].

### 3.5. Membrane Permeation and Retention Performance

Block copolymer membranes with high porosity, homogeneous pore size, and tunable chemical properties hold tremendous potential as robust, efficient, and highly selective separation membranes. We tested the integral asymmetric PS_71_-*b*-P4VP_26_-*b*-PGMA_3_^145^ membrane for ultrafiltration and analyzed the separation performance of the hexagonally organized isoporous structure by comparing it with pristine PS_71_-*b*-P4VP_26_-*b*-PSMA_3_^145^ membrane. The permeance of the membranes before and after hydrolysis was measured in dead-end mode at 2 bar trans-membrane pressure. It is reported in the literature that improvement of hydrophilicity has an influence on the pure water flux [54]. The initial permeance of the pristine PS_71_-*b*-P4VP_26_-*b*-PSMA_3_^145^ and hydrolyzed PS_71_-*b*-P4VP_26_-*b*-PGMA_3_^145^ membrane is 390 ± 25 L m^−2^ bar^−1^ h^−1^ and 485 ± 10 L m^−2^ bar^−1^ h^−1^, respectively. Compared to the pristine membrane, the hydrolyzed membrane decorated with hydrophilic PGMA not only has a higher flux, but also appears to have stable permeance. The pore diameters of both the membranes before and after hydrolysis are rather similar (Figure 4). These increased permeability results are in accordance with the contact angle measurements, the more hydrophilic surfaces lead to an increased wetting of the porous structure [55]. 

The stimuli responsive behavior of the triblock terpolymer membrane PS_71_-*b*-P4VP_26_-*b*-PGMA_3_^145^ was investigated and proved by the influence of the pH value on water flux. Since P4VP can be protonated at low pH, due to the swelling of the positively charged P4VP blocks, a decrease in the size of the pores was observed whereas the highest water flux was observed at pH 7. The P4VP block behaves like a polyelectrolyte at low pH (Supporting Information Figure S7).

Ultrafiltration membranes suffer from organic and biological fouling, which impede the performance in long-term use [56,57,58]. For studying the membrane behavior, retention and adsorption measurements were carried out. The retention measurements were carried out by using 1 mg/1 mL bovine serum albumin (BSA, Sigma-Aldrich, USA) solution at pH 7.4. The isoelectric point is at pH 5.2. BSA shows a negative overall charge at the given pH when taking into account the isoelectric point of the protein. The hydrodynamic diameter of BSA (7.6 nm as stated by the supplier) is much below the pore size; its retention depends mainly on the interaction with the membrane, rather than being a size effect. The results in Table 3 show that the retention rate of BSA for PS_71_-*b*-P4VP_26_-*b*-PSMA_3_^145^ membrane was 90% whereas only 24% retention was observed for hydrolyzed PS_71_-*b*-P4VP_26_-*b*-PGMA_3_^145^ membrane.

**Figure 8:**
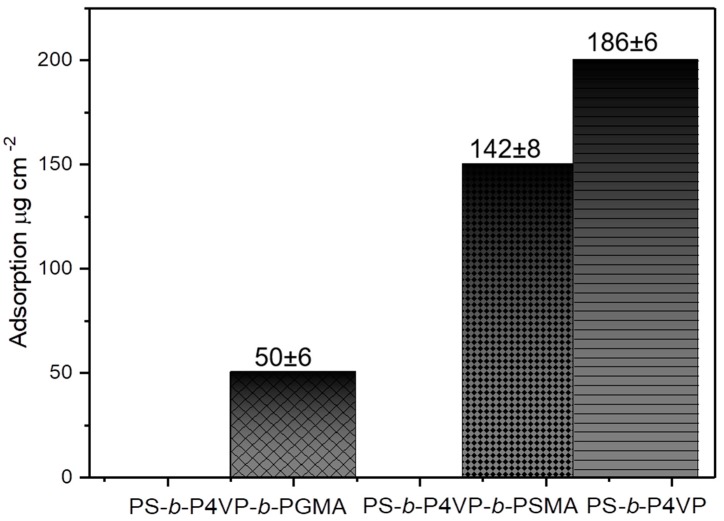
Protein adsorption of hemoglobin of PS_74_-*b*-P4VP_26_^162^, PS_71_-*b*-P4VP_26_-*b*-PSMA_3_^145^, and PS_71_-*b*-P4VP_26_-*b*-PGMA_3_^145^ membranes at pH 7.4.

The low retention rate to the protein demonstrates that increasing the membrane hydrophilicity by incorporating hydroxyl groups containing PGMA results in a decreased fouling [60]. Further on, the high hydrophilicity and the antifouling behavior of PS-*b*-P4VP, pristine PS_71_-*b*-P4VP_26_-*b*-PSMA_3_^145^, and modified PS_71_-*b*-P4VP_26_-*b*-PGMA_3_^145^ membranes were confirmed by static hemoglobin adsorption experiments at 25 °C in PBS buffer (pH 7.4). The hydrodynamic diameter of hemoglobin (IEP = 6.8) is 6.4 nm as stated by the supplier. The results of the adsorption experiments are shown in Figure 8. The modified surfaces were tested in comparison to the untreated triblock terpolymer surfaces. The highest hemoglobin adsorption found for PS_74_-*b*-P4VP_26_^162^ membranes can be attributed to chelation of the iron ions from hemoglobin by the free electron pair of the P4VP nitrogen atom [61,62], whereas a decrease in adsorption value was observed when P4VP pores were covered with a hydrophobic PSMA block. At the given pH value, hemoglobin is not significantly charged (IEP = 6.8) and the membranes are also neutral, which allows the adsorption of hemoglobin to occur because of the hydrophobic/hydrophilic balance of the membranes. PS-*b*-P4VP-*b*-PGMA membranes decorated with hydrophilic PGMA block showed the smallest hemoglobin adsorption among all tested membranes due to strong hydration of the surface, which gives the surface an antifouling property. A remarkable decrease in the adsorption of protein value corresponds to an increase in hydrophilicity of the membrane. The hydrophilic membrane surfaces form hydration layers, which may reduce the adhesion force between the membrane and protein [63]. This significant improvement in the protein adsorption resistance has a profound effect on the long-term filtration of proteins. The antifouling property of PS-*b*-P4VP-*b*-PGMA membranes will be investigated in the future.

## 4. Conclusions

PS-*b*-P4VP-*b*-PSMA triblock terpolymers were synthesized by sequential living anionic polymerization of styrene, 4-vinyl pyridine, and solketal methacrylate with low dispersity values (Ð = 1.06–1.12). If the hydrophobic PSMA block was not too long with respect to the P4VP block, isoporous integral asymmetric membranes could be prepared successfully by SNIPS by using the ternary solvent system (THF/DMF/acetone) from this novel triblock terpolymer. The PSMA block could be successfully converted into a poly(glyceryl methacrylate) block by acidic hydrolysis without affecting the membrane structure. Thus, the introduction of a highly functional and hydrophilic block containing repeating units with two hydroxyl groups could be carried out, which allows for a large variety of further post-modification reactions. The new functional triblock terpolymer PS-*b*-P4VP-*b*-PGMA showed significantly lower retention and fouling compared to the more hydrophobic PS-*b*-P4VP-*b*-PSMA precursor membrane and also compared to a PS-*b*-P4VP membrane, indicating a strong and positive influence of the third hydrophilic block on the membrane properties.

## Figures and Tables

**Table 1:** Composition and molecular weights of polystyrene-*b*-poly(4-vinylpyridine)-*b*-poly(solketal methacrylate) (PS-*b*-P4VP-*b*-PSMA) triblock terpolymers in this study.

Polymers ^a,b^	*M*_n_ (kg/mol)	Ð
PS_71_-*b*-P4VP_26_-*b*-PSMA_3_^145^	145	1.06
PS_70_-*b*-P4VP_25_-*b*-PSMA_5_^143^	143	1.03
PS_71_-*b*-P4VP_17_-*b*-PSMA_12_^91^	91	1.12

^a^ Subscript shows the weight percentages of individual blocks calculated from ^1^H-NMR. ^b^ Superscript shows the total molecular weight of polymer.

**Table 2:** Hansen solubility parameters (δ) of homopolymers, solvents, and non-solvents [51].

Polymer	δ_D_ (MPa^0.5^) ^a^	δ_P_ (MPa^0.5^) ^a^	δ_H_ (MPa^0.5^) ^a^	δ = √ δ_D_^2^ + δ_P_^2^ + δ_H_^2^
PS	18.5	4.5	2.9	19.3
P4VP	18.1	7.2	6.8	19.0
THF	16.8	5.7	8.0	19.5
DMF	17.4	13.7	11.3	24.8
DOX	17.5	1.8	9.0	19.8
Acetone	15.5	10.4	7.0	19.9
Water	15.6	16.0	42.3	47.8
**Solubility Parameters by the Hoy Method [52]**				
PSMA	16.76	9.35	5.54	19.9
PGMA	19.25	9.23	14.4	25.8

^a^ Dispersion solubility parameter; δ_D_, polar solubility parameter; δ_P_, hydrogen bonding solubility parameter; δ_H_.

**Table 3:** Retention results from experiments in a 1 mg/1 mL bovine serum albumin (BSA) solution in PBS buffer (pH = 7.4).

Membrane/BSA	% BSA Retention	Average Pore Diameter/Size (nm)
PS_71_-*b*-P4VP_26_-*b*-PSMA_3_^145^	90	26 ± 3 ^a^
PS_71_-*b*-P4VP_26_-*b*-PGMA_3_^145^	24	26 ± 4 ^a^
BSA	-	7.6 [59]

^a^ Determined by analyzing the SEM surface images.

## Supplementary Materials

The following are available online at https://www.mdpi.com/2073-4360/12/1/41/s1, Figure S1: SEM images of PS_71_-*b*-P4VP_26_-*b*-PSMA_3_^145^ membrane surfaces prepared from different solutions: 22 wt% copolymer in (a) 60/40 THF/DMF; (b) 50/50 THF/DMF; (c) 70/30 THF/DMF. The evaporation time before immersion into the precipitant was 10 seconds for the three different concentrations. Figure S2. SEM images of the surfaces of PS_71_-*b*-P4VP_26_-*b*-PSMA_3_^145^ membranes cast from a 22 wt % copolymer solution in (a) THF/DMF/DOX 1/1/1 (b) THF/DMF/DOX 40/30/30. The evaporation time before immersion into the precipitant was 10 seconds. Figure S3. SEM images of the surfaces of PS_71_-*b*-P4VP_26_-*b*-PSMA_3_^145^ membranes cast from solutions THF/DMF/Acetone: 50/30/20 wt%. (a) 20 seconds (b) 30 seconds evaporation time before immersion into non-solvent bath. Figure S4. SEM images of the surfaces of PS_71_-*b*-P4VP_26_-*b*-PSMA_3_^145^ membranes cast from 21 wt%, 23wt%, 24wt% copolymer solutions in THF/DMF/Acetone: 50/30/20 wt%. The evaporation time before immersion into the precipitant was 10 seconds. Figure S5. SEM images of the surface of (a) pristine PS_70_-*b*-P4VP_25_-*b*-PSMA_5_^143^ membrane and (b) PS_70_-*b*-P4VP_25_-*b*-PGMA_5_^143^ membrane after acidic hydrolysis. The evaporation time before immersion into water bath was 10 seconds. Figure S6. SEM images of (a) PS_71_-*b*-P4VP_17_-*b*-PSMA_12_^91^ membrane (b) PS_71_-*b*-P4VP_17_-*b*-PGMA_12_^91^ membrane obtained after acidic hydrolysis. The evaporation time before immersion into water bath was 10 seconds. Figure S7. Water permeabilities of PS_71_-b-P4VP_26_-*b*-PGMA_3_^145^ membrane measured at various pH, at pH ˃ 4 high water permeability was observed, due to deswelling of the deprotonated P4VP blocks at larger pH, leading to their collapse on the pore walls. Table S1. Comparison of dynamic contact angle values of PS_71_-*b*-P4VP_26_-*b*-PSMA_3_^145^ and PS_71_-*b*-P4VP_26_-*b*-PGMA_3_^145^ triblock terpolymer membranes.
