# Supplementary material for: Isoporous Membranes from Novel Polystyrene-b-poly(4-vinylpyridine)-b-poly(solketal methacrylate) (PS-b-P4VP-b-PSMA) Triblock Terpolymers and Their Post-Modification
Source: Polymers (Basel). 2019 Dec 26;12(1):41. doi: 10.3390/polym12010041 (PMC7023574; doi:10.3390/polym12010041)
Supplement: Supplementary file 1 [file polymers-12-00041-s001.pdf]

polymers

MDPI

Supporting Information

Isoporous Membranes from Novel polystyrene-*b*-poly(4-vinylpyridine)-*b*-poly(solketal methacrylate) (PS-*b*-P4VP-*b*-PSMA) Triblock Terpolymers and their Post-modification

Sarah Saleem[1], Sofia Rangou[1], Clarissa Abetz[1], Volkan Filiz[1], Volker Abetz[1,2*]

1. Helmholtz-Zentrum Geesthacht, Institute of Polymer Research, Max-Planck-Str.1, 21502 Geesthacht, Germany.
2. Universität Hamburg, Institute of Physical Chemistry, Martin-Luther-King-Platz 6, 20146 Hamburg, Germany.
* Correspondence: E-Mail: volker.abetz@hzg.de, Tel: +49 4152 872461

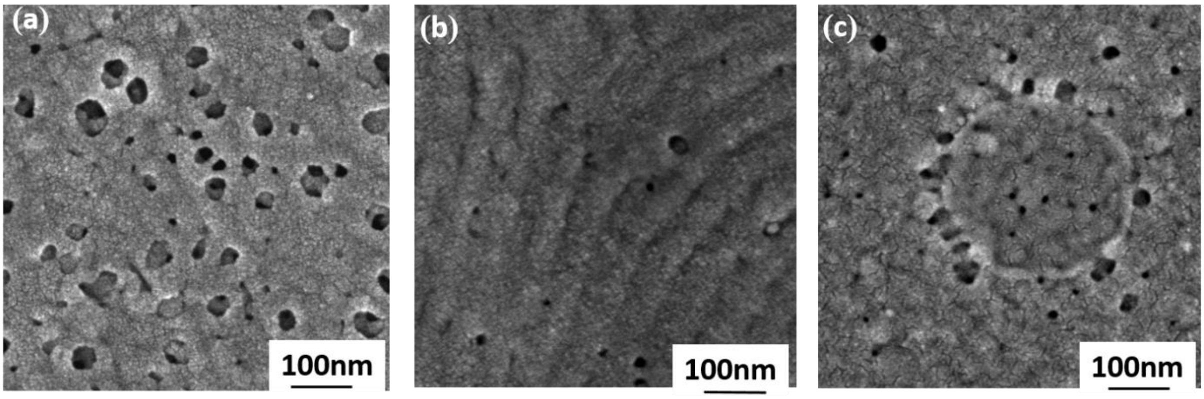

Figure 1. SEM images of PS_{71}-*b*-$P4VP_{26}$-*b*-$PSMA_{3}^{145}$ membrane surfaces prepared from different solutions: 22 wt% copolymer in (a) 60/40 THF/DMF; (b) 50/50 THF/DMF; (c) 70/30 THF/DMF. The evaporation time before immersion into the precipitant was 10 seconds for the three different concentrations.

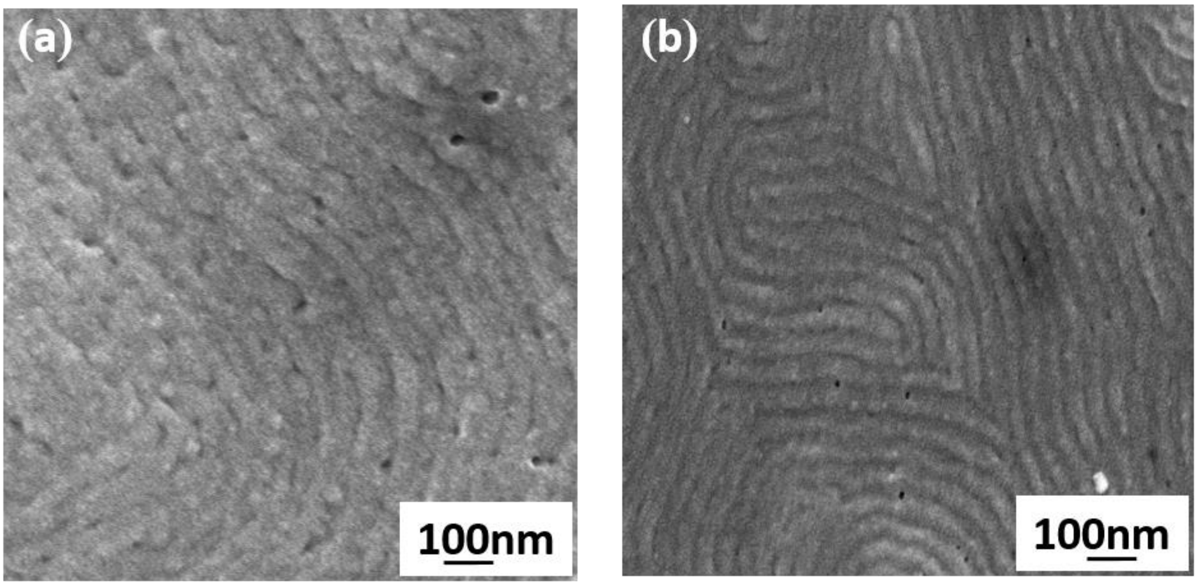

Figure 2. SEM images of the surfaces of PS_{71}-*b*-$P4VP_{26}$-*b*-$PSMA_{3}^{145}$ membranes cast from a 22 wt% copolymer solution in (a) THF/DMF/DOX 1/1/1 (b) THF/DMF/DOX 40/30/30. The evaporation time before immersion into the precipitant was 10 seconds.

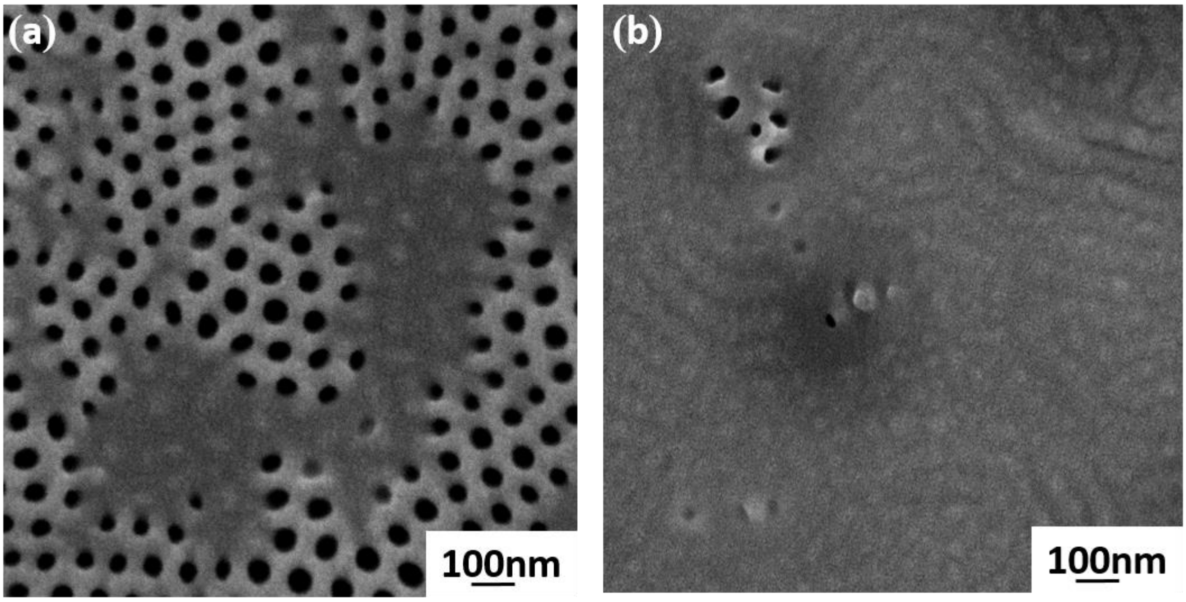

Figure 3. SEM images of the surfaces of PS_{71}-*b*-$P4VP_{26}$-*b*-$PSMA_{3}^{145}$ membranes cast from solutions THF/DMF/Acetone: 50/30/20 wt%. (a) 20 seconds (b) 30 seconds evaporation time before immersion into non-solvent bath.

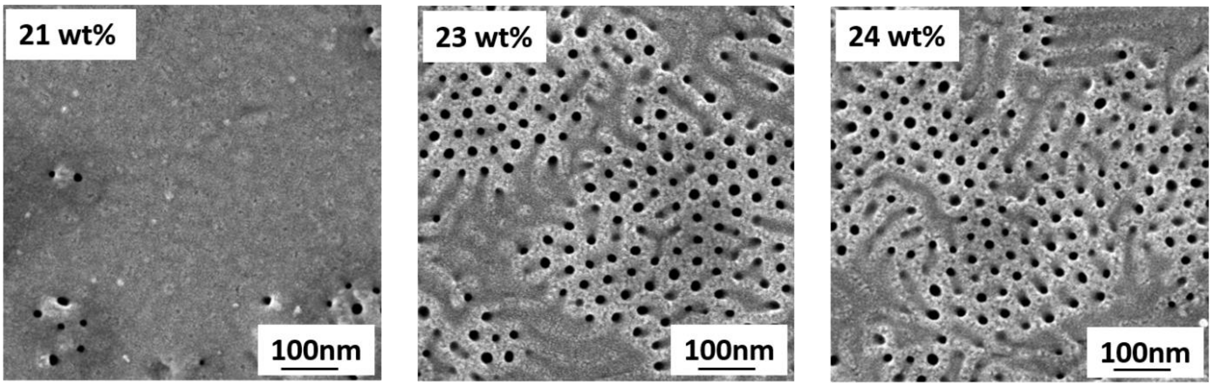

Figure 4. SEM images of the surfaces of PS_{71}-*b*-$P4VP_{26}$-*b*-$PSMA_{3}^{145}$ membranes cast from 21 wt%, 23wt%, 24wt% copolymer solutions in THF/DMF/Acetone: 50/30/20 wt%. The evaporation time before immersion into the precipitant was 10 seconds.

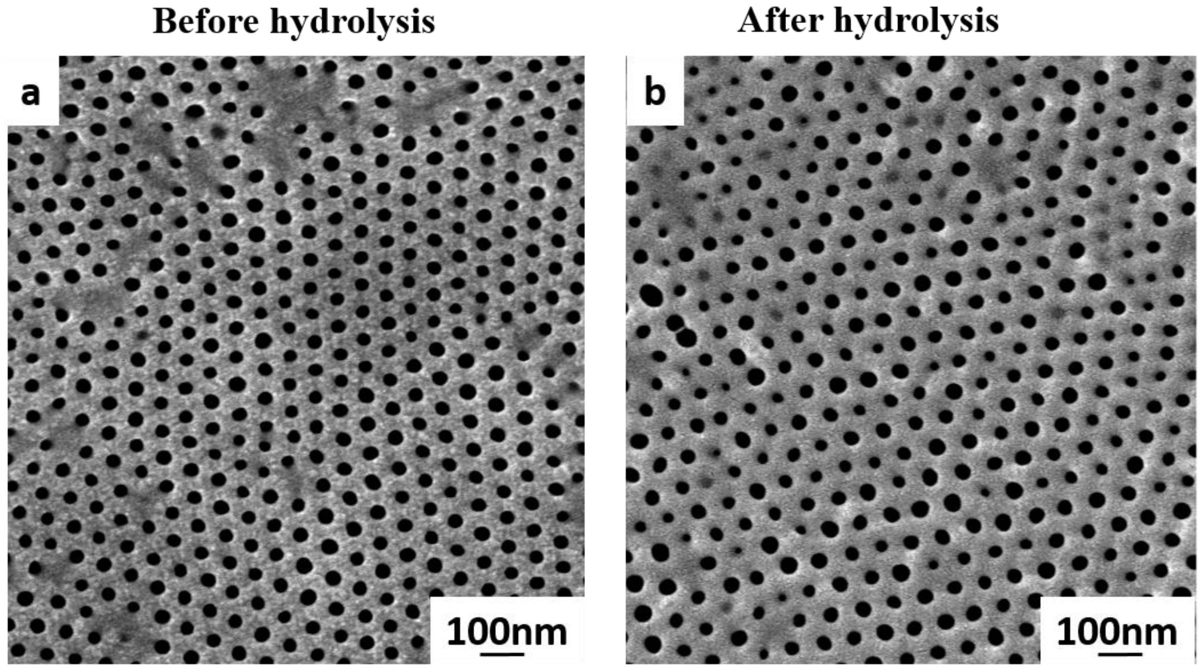

Figure 5. SEM images of the surface of (a) pristine PS_{70}-*b*-$P4VP_{25}$-*b*-$PSMA_{5}^{143}$ membrane and (b) PS_{70}-*b*-$P4VP_{25}$-*b*-$PGMA_{5}^{143}$ membrane after acidic hydrolysis. The evaporation time before immersion into water bath was 10 seconds.

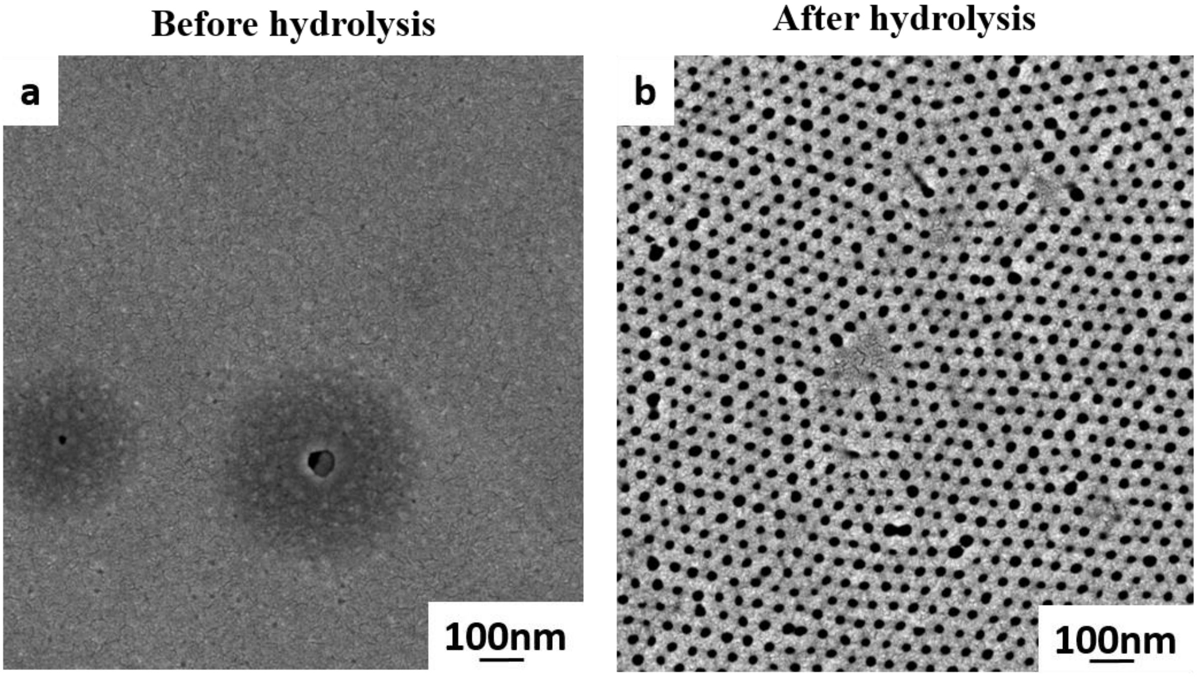

Figure 6. SEM images of (a) PS_{71}-*b*-$P4VP_{17}$-*b*-$PSMA_{12}^{91}$ membrane (b) PS_{71}-*b*-$P4VP_{17}$-*b*-$PGMA_{12}^{91}$ membrane obtained after acidic hydrolysis. The evaporation time before immersion into water bath was 10 seconds.

Table 1. Comparison of dynamic contact angle values of PS_{71}-*b*-$P4VP_{26}$-*b*-$PSMA_{3}^{145}$ and PS_{71}-*b*-$P4VP_{26}$-*b*-$PGMA_{3}^{145}$ triblock terpolymer membranes.

PS_{71}-*b*-$P4VP_{26}$-*b*-$PSMA_3^{145}$ (°)	PS_{71}-*b*-$P4VP_{26}$-*b*-$PGMA_3^{145}$ (°)
63±1	51±1
61±0.5	47±3
56±2	38±2
49±1	37±3
45±1	34±3
43±0.46	27±3
39±3	22±1
34±2	17±2
30±1	13±1
25±4	9±1
16±5	

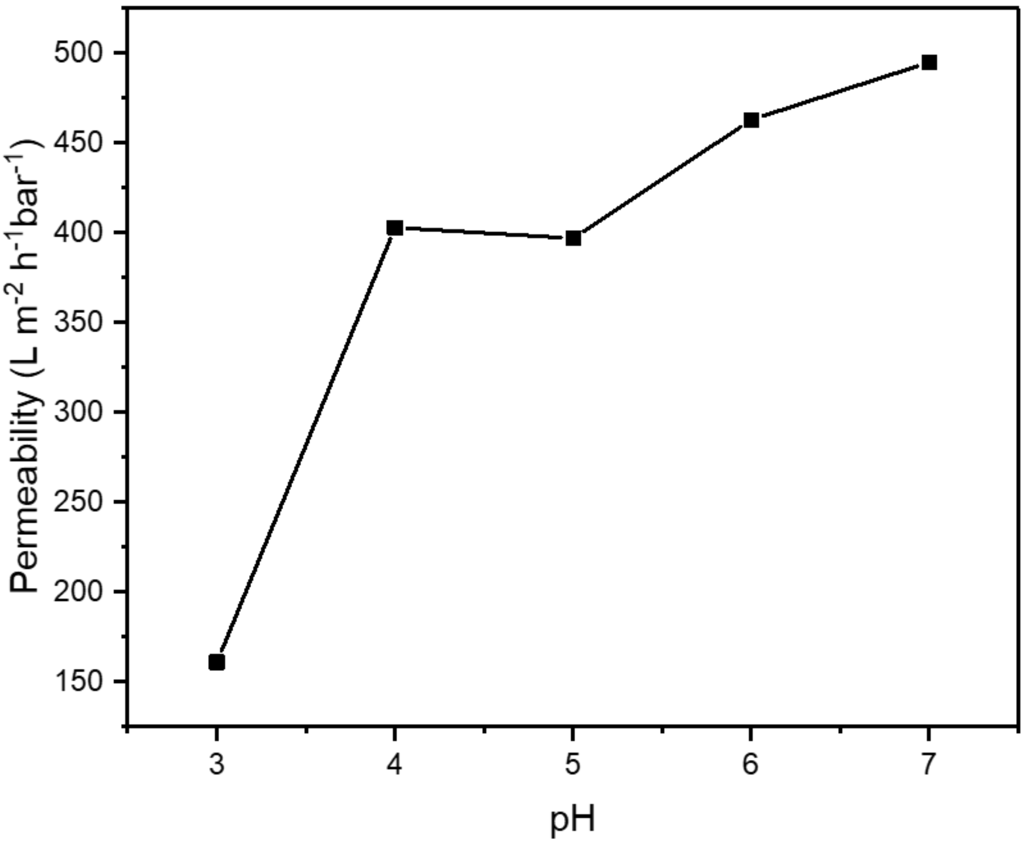

Figure 7. Water permeabilities of PS_{71}-*b*-$P4VP_{26}$-*b*-$PGMA_3^{145}$ membrane measured at various pH, at pH > 4 high water permeability was observed, due to deswelling of the deprotonated P4VP blocks at larger pH, leading to their collapse on the pore walls.
